# Detoxification of Grape Pomace Contaminated with Ochratoxin A by Thermal–Pressure Treatment in Combination with Lactic Acid Bacteria Fermentation

**DOI:** 10.3390/microorganisms13091972

**Published:** 2025-08-23

**Authors:** Ebenezer Aning-Dei, Jianmei Yu, Salam A. Ibrahim

**Affiliations:** Food and Nutritional Sciences, North Carolina Agricultural and Technical State University, 1601 East Market Street, Greensboro, NC 27411, USA

**Keywords:** grape pomace, ochratoxin A, polyphenols, pressure cooking, fermentation, lactic acid bacteria

## Abstract

Grape pomace (GP), a polyphenol-rich byproduct of winemaking, holds considerable health benefits and potential as an antibiotic alternative for livestock animals. However, its utilization is compromised by the contamination of mycotoxins produced by pathogenic molds (with ochratoxin A (OTA) being the most frequently detected), which pose hidden health risks to both livestock animals and human beings. This study evaluated the efficacy of thermal–pressure treatment (pressure cooking) with and without the addition of acidic and alkaline agents, and the combined thermal-pressure and fermentation with four lactic acid bacteria (LAB) strains, including *Lactobacillus bulgaricus (LB6)*, *Lacticaseibacillus paracasei* (previously *Lactobacillus paracasei) (BAA-52)*, *Lactobacillus acidophilus*, *and Lactiplantibacillus plantarum* (previously *Lactobacillus plantarum*), on reducing OTA and preserving polyphenols in GP. The study found that pressure cooking alone reduced OTA by approximately 33–35% in 30–45 min. The addition of citric acid (CA) or acetic acid (AA) enhanced OTA reduction to 46.9–55.2% and 51.7–54%, respectively, while preserving more polyphenols, notably anthocyanins. Conversely, pressure cooking with the addition of NaHCO_3_ facilitated greater OTA reductions (40.4–63%), but concomitantly resulted in substantial polyphenol loss, especially anthocyanins. Fermentation for 24 h with LAB following thermal–pressure treatment resulted in up to 97% OTA reduction for *Lc. paracasei*, *L. acidophilus,* and *Lp. plantarum* strains, which displayed similar high effectiveness in OTA reduction in GP. *L*. *bulgaricus* (*LB6*) was least effective (45%), even after 72 h of fermentation. These findings indicate that home-scale pressure cooking combined with lactic acid fermentation effectively detoxifies OTA-contaminated GP, thus enhancing its safety profile for consumption by livestock animals and humans, despite partial polyphenolic losses.

## 1. Introduction

Grape pomace (GP) is a valuable dietary fiber-rich byproduct of the wine industry, consisting of the skins, seeds, stems, and pulp of grapes after juice or wine extraction. Approximately 13 million tons of grape pomace are produced annually worldwide, predominantly in wine-producing countries like Italy, France, Spain, and the United States, and typically discarded as waste or limited to fertilizer and biofuel uses [1,2]. However, recent research highlights its bioactive compounds, such as polyphenols, which exhibit anti-inflammatory and strong antioxidant properties, offering protective effects against oxidative stress and biomolecular damage [3,4].

Beyond their antioxidant capacity, polyphenols from grape pomace (GP) possess antimicrobial properties, making them promising natural alternatives to antibiotics in animal nutrition by enhancing immune health [5]. Studies indicate that GP inclusion in animal feed at a proper level improves feed efficiency, meat quality, and egg production, particularly in poultry and swine [6,7]. Specifically, GP positively influences poultry gut microbiota, boosting beneficial bacteria while suppressing harmful strains like *Escherichia coli* and *Salmonella* [8,9]. In human nutrition, incorporating GP into baked products significantly boosts dietary fiber and polyphenol content, although consumer acceptance of cookies declined at higher GP levels [10,11,12]. Overall, GP’s polyphenols and dietary fibers support gut health by modulating the gut microbiota and enhancing intestinal barrier function, offering functional benefits to both humans and livestock. However, limited studies have found that GP is often contaminated with high level of ochratoxin A (OTA), a toxic secondary metabolite of pathogenic molds such as *Aspergillus* and *Penicillium* species [13,14,15]. OTA poses significant health risks to both humans and animals. OTA has been reported to be nephrotoxic, hepatotoxic, immunotoxic, neurotoxic, teratogenic, and carcinogenic in many animal studies [16]. Animals fed with OTA-contaminated feed showed reduced performance and had higher OTA residues in their meat and organs, a hidden health risk to consumers that can cause a range of diseases including liver damage, immune suppression, and renal diseases [16]. The presence of OTA in GP thus limits its safe use as a food and feed ingredient.

To protect human and livestock animals from OTA toxification, many countries, particularly those within the European Union (EU), have established and implemented regulations for different food categories and feeds. In the EU, the maximum limits for OTA in human food products vary from 0.5 µg/kg in infant formula to 5 µg/kg in unprocessed grains, 3 µg/kg in processed cereal products, 2 µg/kg in wine and juice, 10 µg/kg in roasted coffee and dry fruits, and 20 µg/kg in spices, while the regulation limits in feeds are 50 µg/kg for swine and 100 µg/kg for poultry [17]. Canada’s proposed OTA regulatory guide mirrors the EU’s standards [18]. As of now, the United States Food and Drug Administration (FDA) has not set regulatory standards for OTA in food or feed.

Several detoxification methods, including irradiation, heat treatment, fermentation, enzymatic, oxidizing agents, and alkaline and acid treatments, have been studied to reduce OTA levels in food products [15,19,20]. The efficacy of heat treatment on OTA reduction significantly depends on pH, temperature, and processing duration. For instance, OTA levels decreased by over 90% at 200 °C under most pH conditions, except at pH 4. Alkaline conditions (pH 10) at 100 °C for 60 min led to about 50% OTA reduction, while acidic and neutral conditions at the same settings were less effective [20]. However, thermal degradation of OTA under alkaline conditions (pH 10) could form a more toxic open-ring form (OP-OTA) [20]. The degradation products are also affected by the food matrix. The main thermal degradation product is 2′R-Ochratoxin A (2′R-OTA) in the absence of sugar, whereas OTα and OTα-amide are mainly produced in the presence of sugar [21]. OTA is generally heat-stable, requiring prolonged exposure at high temperatures for significant degradation, such as over 10 h at 100 °C to achieve 50% reduction in dry wheat [22]. Higher temperatures, such as those used in coffee roasting, substantially reduced OTA (8–98%) [23], but roasting is not suitable for grape pomace detoxification due to the risk of carbonization.

Pressure cooking is a food processing method that effectively reduces microbial contaminants and harmful substances but cannot achieve a high OTA reduction, as previously reported [15]. Certain bacteria, including *Bacillus subtilis* and lactic acid bacteria, have demonstrated a strong ability to significantly reduce OTA by adsorption and physical removal or enzymatic transformation of OTA into non-toxic or less toxic compounds such as OTα and OTα-amide [19]. In addition, the fermentation is often conducted anaerobically at mild temperatures (~40 °C), which is essential for polyphenol retention. Therefore, combining pressure cooking with acid/alkaline treatments or fermentation might enhance OTA detoxification in GP. However, the effectiveness of fermenting GP specifically, given its high polyphenol and fiber but low fermentable sugar contents, remains unknown. This study aims to evaluate the effects of pressure cooking combined with acid/alkaline treatments and *lactobacillus* fermentation on OTA levels and polyphenol preservation in GP. This research could enhance food safety and sustainability and add economic value to winery byproducts.

## 2. Materials and Methods

### 2.1. Materials

Grape Pomace (GP) from the blend of Merlot and petite Verdot grapes was collected from a local winery (Gibsonville, NC USA) right after the pressing of the fermented marc and stored in the freezer at −20 °C for later use. Amber glass bottles, spatulas, weighing boats, conical flasks, centrifuge tubes, syringes, amber vials, test tubes, and a strainer were purchased from Fisher Scientific. Pipette tips were from Eppendorf (Enfield, CT, USA). Freeze-dried cultures of lactic acid bacteria (LAB) strains—*Lacticaseibacillus paracasei (BAA-52)*, *L. bulgaricus (LB6)*, *L. acidophilus* (ATCC 4356), *and Lactiplantibacillus plantarum* (ATCC 10241)—were purchased from American Type Culture Collection (ATCC, Manassas, Virginia, USA) and kept at 4 °C until use. The *L. bulgaricus (LB6)* was isolated from commercial Bulgarian yogurt by a scientist who previously worked in our department. The UV visible spectrophotometer was from Thermo Fisher Scientific (Waltham, MA, USA). Incubator (3193 Infrared CO_2_, Forma Scientific, Marietta, OH, USA).

HPLC-grade acetonitrile (Alfa Aesar, Ward Hill, MA), sodium hydroxide (NaOH), hydrochloric acid (HCl), acetic acid, citric acid, and sodium bicarbonate (NaHCO_3_) were purchased from Fisher Scientific (Hampton, NH, USA). Ochratoxin A standard solution and OTA powder, gallic acid, catechin, and Folin–Ciocalteu reagent were purchased from Sigma-Aldrich (St. Louis, MO, USA). HPLC-grade methanol was purchased from Avantor VWR International (Suwanee, GA, USA). De Man, Rogosa, and Sharpe (MRS) culture media was purchased from Neogen (Lansing, MI, USA).

### 2.2. Preparation of Grape Pomace (GP)

The grape pomace (GP) was thawed at room temperature after removal from the freezer. To reduce tannin content, the free seeds were manually removed. During the optimization of OTA extraction and analysis before any detoxification treatment, the OTA was not detected from the GP collected. The GP was then artificially contaminated with OTA solution (Sigma-Aldrich). A working OTA solution of 1 µg/mL was prepared in methanol from an OTA stock solution (10 µg/mL). Five 50 g portions of GP were weighed into amber glass bottles, spiked with 5 µL of 100 µg/mL OTA—resulting in an OTA concentration of 10 ng/g GP for each portion, the maximum allowance of OTA in processed grape-based foods such as dried vine fruits set by European Union [24]—mixed, and capped. Samples were subjected to thermal treatment in a pressure cooker at 12 psig and 115 °C for 15, 30, 45, and 60 min, respectively. Control samples were spiked with OTA but not pressure cooked. For each treatment time, experiments were carried out in triplicate under identical conditions. During treatments, samples were not interrupted before reaching the designated treatment time.

### 2.3. Pressure Cooking of Grape Pomace with Added Acid

Citric acid (CA) and acetic acid (AA) stock solutions of 1000 mM were prepared from solid CA and glacial AA (99.9% purity), respectively, and the 100 mM and 10 mM acid solutions were prepared by serial dilution of the 1000 mM stock solution with deionized (DI) water. The artificially contaminated GP with OTA was divided into six portions (each 50 g) and placed in six amber glass bottles; 50 mL of acid solution (10, 100, and 1000 mM) was added to each bottle. The bottles were capped after mixing with a spatula. The OTA-spiked GP sample without the addition of acid was used as control. All samples were pressure cooked for 45 min at 12 psig and 115 °C without interruption. The samples were allowed to cool to room temperature after the treatment for extraction. All experiments were carried out in triplicate.

### 2.4. Pressure Cooking of Grape Pomace with Added Alkaline

A 100 mM sodium bicarbonate (NaHCO_3_) solution was prepared from powder by dissolving 8.4 g in 1 L DI water. A volume of 100 mL of the NaHCO_3_ solution was added to 50 g of the GP artificially contaminated with OTA solution to 10 ng/g, mixed, and the pH adjusted to 5, 6, and 7 with 2 N sodium hydroxide (NaOH) or 2 N hydrochloric acid (HCl). The OTA-spiked GP sample without NaHCO_3_ and pH adjustment was used as a control. All samples were spiked to 10 ng OTA/g GP with OTA solution and pressure cooked at 12 psig and 115 °C for 15, 30, 45, and 60 min at each pH level. Samples in different pH conditions were treated for the same amount of time. All experiments were carried out in triplicate under identical conditions.

### 2.5. Bacteria Culture Activation and Preparation

LAB strains were purchased from (ATCC, Manassas, VA, USA) and stored in the stock collection of the Food Microbiology and Biotechnology Laboratory, North Carolina A&T State University. The strains were activated in MRS by transferring 100 µL of the stock culture to 10 mL MRS broth, incubated at 42 °C for 12 h, and stored at 4 °C. Prior to each experimental replication, bacterial strains were streaked on MRS agar and incubated for 48 h at 42 °C. One isolated colony was then transferred to 10 mL MRS broth and incubated at 42 °C for use the following day.

### 2.6. Semi-Solid Fermentation of Grape Pomace

A 5 × 6 two-factor factorial design was used to determine the right bacterial strains and optimal fermentation time. Due to the reported high OTA reduction by fermentation, the GP for fermentation experiment was spiked to 50 ng OTA per gram GP (50 ng/g) and mixed thoroughly to ensure that the residue OTA could be detected after fermentation. Before fermentation, 50 mL of NaHCO_3_ and 4 g of sucrose was added to 50 g of OTA-spiked GP, pH adjusted to 6.5, the optimal pH for LAB growth, with 1 N NaOH and 1 N HCl, and pressure cooked for 45 min. After pressure cooking, the GP samples were cooled to room temperature and then inoculated with one of the four (4) LAB strains for fermentation. This was performed in triplicate for each strain. The LAB strains were activated in 3 mL De Man, Rogosa, and Sharpe (MRS) broth overnight. A volume of 100 µL of each activated strain was re-cultured in 10 mL of fresh MRS for 12 h in the incubator to achieve optical densities (ODs) of 0.7–0.9 at 610 nm. Each re-cultured strain was mixed with 15 g of sterile GP in a bottle and kept in an incubator at 42 °C for 2 h for adaptation, and then 1.5 mL of each adapted cultured strain was pipetted and added to 50 g of pressure cooked GP sample, which resulted in a bacterial concentration of 10^8^ CFU/g GP. All samples were mixed, capped, and placed in an airtight container, and fermentation was conducted in the incubator (3193 Infrared CO_2_, Forma Scientific, Marietta, OH, USA) at 42 °C for 4, 8, 12, 24, 48, and 72 h. The pH was recorded at the beginning and end of each fermentation duration. The pressure-cooked OTA-spiked GP served as a control for fermentation (0 fermentation time). After fermentation, OTA extraction and quantification were carried out according to the method described in Section 2.8.

### 2.7. Bacteria Enumeration

The bacterium concentration in GP at each sampling time was determined by the plating method using MRS agar plates. Peptone water (0.1%) was used for bacterium extraction. Briefly 15 g of the fermented grape pomace at each fermentation time was mixed with 100 mL of peptone water for 2 min using a Stomacher^®^ 80 Biomaster (Seward Laboratory Systems, Inc., Worthing, UK). Then, 1 mL of the mixture was pipetted and serially diluted in 9 mL of 0.1% peptone water, and each dilution was plated on MRS agar plates and incubated at 42 °C for 48 h. The procedure was performed in triplicate for each sample. The experiment was carried out in a biosafety cabinet. The number of colonies on each plate was counted with the UVP ChemStudio Imaging System (Analytik Jena, CA, USA). Bacterial colonies were expressed in log CFU/mL by

(1) $$CFU/mL=\frac{Number\;of\;colonies\;\times \;Dilution\;factor}{Volume\;of\;culture\;per\;plate},$$

### 2.8. Sample Extraction and Analysis

After pressure cooking and cooling, the GP samples were homogenized for 1–3 min using a high-speed blender (Fisher Scientific, USA) to make a slurry. An amount of 10 g of the slurry was mixed with 20 mL undiluted methanol in a 50 mL conical flask and stirred for 30 min. The mixture was then centrifuged at 3000 rpm for 20 min using an Eppendorf 5810R Centrifuge (Enfield, CT, USA), and the supernatant was collected into clean storage tubes and stored at −20 °C for OTA, total anthocyanin (TA), total flavonoid (TF), and total polyphenol (TP) quantification. The procedure was repeated for all samples and analyses were performed in triplicate.

#### 2.8.1. OTA Quantification and Recovery

The clear supernatants collected were transferred into UPLC amber vials and analyzed for OTA using an Agilent 1260 Infinity II UPLC system (Santa Clara, CA, USA) with a Fluorescence detector operating at an excitation wavelength of 330 nm and emission wavelength of 470 nm. The OTA was separated in a Kinetex C18 Column (150 × 4.6 mm, 5 um) (Phenomenex, Torrance, CA, USA) using a mobile phase composed of acetonitrile, water, and acetic acid in ratio of 49.5:49.3:1.2 under isocratic mode. The sample injection volume was 20 µL, and the mobile phase flow rate was 0.8 mL/min. The peak area and retention time from the chromatogram for the samples were recorded, and the OTA concentration was calculated from the standard calibration curve developed under the same chromatography condition as that for samples. The OTA standard concentrations were in the range of 0.25–50 ng/mL. The OTA in GP sample extracts was identified based on the retention time. The analysis was performed in triplicate for each sample.

For the OTA recovery test, the deseeded GP was spiked with OTA stock solution to concentrations of 10 ng/g GP and 50 ng/g GP, respectively, in the bottles, mixed, capped, and then placed at 4 °C overnight. The deseeded GP without OTA spiking was used as control. The OTA recovery rate was determined using spiked GP under the OTA extraction and analysis conditions used in this study. For each spiking level, the recovery test was conducted in triplicate. The OTA recovery rate was calculated as follows:

(2) $$Recovery(\%)=\frac{Measured\;Concentration}{Spiked\;Concentration}\times 100$$

The OTA recovery was 75.83–76.75% (average 76.71%) at a 10 ng/g spiking level and 80.05–81.29% (average 80.54%) at a spiking level of 50 ng/kg GP. The OTA data presented in this manuscript were adjusted using these two recovery rates.

#### 2.8.2. Total Anthocyanin (TA) Quantification

The TA content was determined using the pH difference method according to the AOAC method 2005.02 [24]. An appropriate dilution factor was determined by diluting the test portion of the GP sample extract with pH 1.0 buffer until the absorbance at 520 nm was within the linear range of the spectrophotometer (0.2–1.4 AU). Using this dilution factor, two dilutions of the test sample, one with pH 1.0 buffer and the other with pH 4.5 buffer, were prepared for each sample. The absorbances of each diluted extract were measured with a Genesys 10 UV-Vis Spectrophotometer at 520 and 700 nm within 20–50 min of preparation. The blank had all reagents without sample extract. The TA concentration was calculated using the equation below and expressed as cyaniding-3-glucoside (cyd-3-glu) equivalents per 100 g of fresh grape for the triplicate extracts.

Anthocyanin (cyd-3-glu, equivalents, mg/L) = (A × MW × DF × 10^3^)/(ε × L), (3)

where A = (A520 nm–A700 nm) pH 1.0—(A520 nm–A700 nm) pH 4.5, MW (molecular weight) = 449.2 g/mol for cyaniding-3-glucoside (cyd-3-glu), DF = dilution factor established, L = path length in cm, ε= 26,900 molar extinction coefficient, in L *${mol}^{-1}$ *${cm}^{-1}$, for cyd-3-glu, and 10^3^ = factor for conversion from g to mg.

#### 2.8.3. Total Flavonoid (TF) Quantification

The TF concentration was determined as previously described [25]. Briefly, 0.25 mL of the supernatant and 0.075 mL of 5% sodium nitrite solution (NaNO_2_) were mixed in a 10 mL test tube and allowed to react for 6 min. A volume of 0.15 mL (150 µL) of 10% aluminum chloride was added and allowed to react for 5 min. Finally, 0.5 mL of 1 M sodium hydroxide (NaOH) was added. The mixture was brought to a final volume of 2.5 mL with deionized water and mixed using a vortex. The absorbance of the mixture was immediately measured at 510 nm against a prepared blank using a spectrophotometer. The blank was the same mixture without sample extract. The flavonoid content was calculated using a calibration curve developed by using the (+)-catechin standard solutions (10–1000 µg/mL) and expressed as catechin equivalents (mg of CAE/g sample).

#### 2.8.4. Total Polyphenol (TP) Quantification

The TP content was determined using the Folin–Ciocalteu method. Initially, a 10% sodium carbonate (Na_2_CO_3_) solution was prepared by dissolving 10 g of Na_2_CO_3_ in 100 mL of distilled water. For the assay, 20 µL of GP extract was transferred into test tubes in triplicate. Subsequently, 1.28 mL of distilled water and 100 µL of Folin–Ciocalteu (Sigma-Aldrich, St. Louis, MO, USA) reagent were added to each tube. The tubes were capped, mixed thoroughly, and incubated in darkness for 8 min. After incubation, 0.6 mL of the 10% Na_2_CO_3_ solution was added, and the mixtures were again capped, mixed, and incubated in the dark at room temperature for 2 h to allow for color development. Absorbances were recorded at 765 nm using a spectrophotometer. TP concentration was calculated using a standard calibration curve generated using gallic acid solutions with known concentrations. Results were expressed as milligrams of gallic acid equivalents (mg GAE) per 100 g of fresh sample, presented as mean ± standard deviation (SD) from three independent replicates. The whole analysis procedure was conducted under dim light. 

### 2.9. Statistical Analyses

R software (R Foundation for Statistical Computing, Vienna, Austria, version 2024.12.1 + 563) was used to analyze the experimental data obtained in this study. The data were analyzed by two-way ANOVA to assess the main effects and interactions of the factors on OTA levels and polyphenol contents. Post hoc Tukey tests were conducted to determine if there were significant differences between the treatment groups at *p* ˂ 0.05, and regression analysis was conducted to reveal the trends of changes in OTA and polyphenols with treatment time.

## 3. Results

### 3.1. Representative Chromatograms of OTA Standard and Grape Pomace Extracts

**Figure 1:**
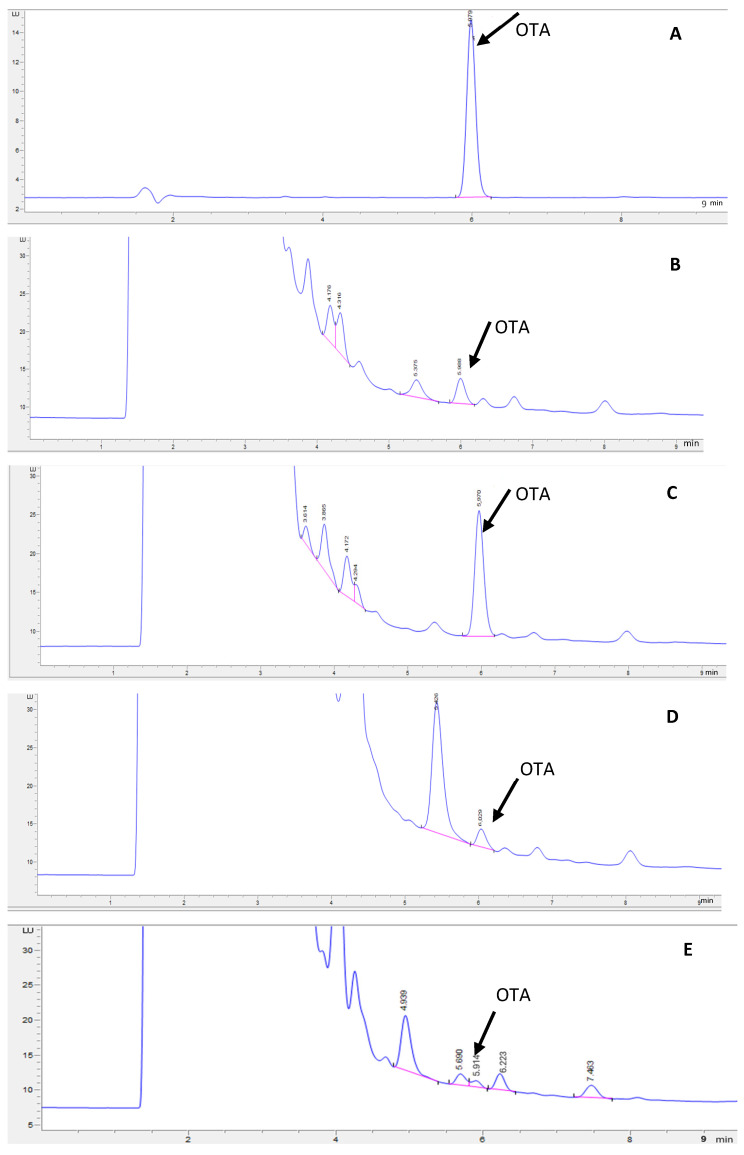

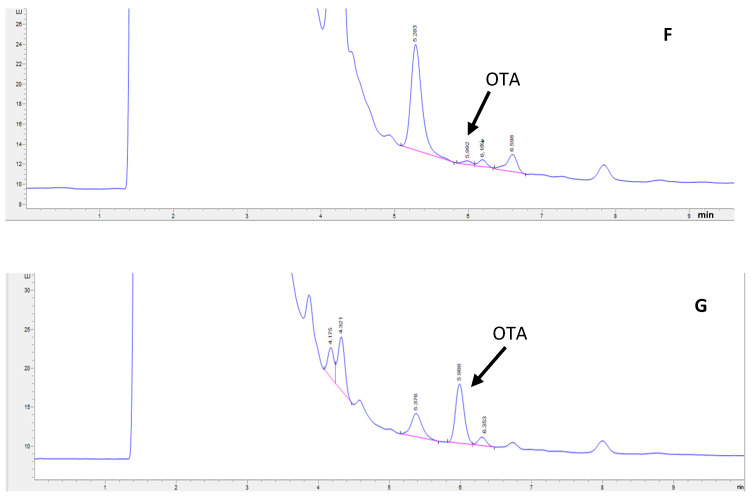
OTA chromatograms of (**A**) OTA standard (10 ng/mL), (**B**) spiked GP (10 ng/g), (**C**) spiked GP (50 ng/g), (**D**) OTA-spiked (10 ng/mL) and pressure-cooked GP at pH 4.3 (30 min), (**E**) OTA-spiked (10 ng/mL) and pressure-cooked GP at pH 7 (30 min), (**F**) *L. acidophilus*-fermented GP (24 h), and (**G**) *L. bulgaricus-fermented* GP (24 h).

The UPLC chromatogram of the OTA standard and the representative chromatogram of extracts of OTA-spiked GP samples without and with fermentation are given in Figure 1. The calibration equation obtained within an OTA range of 0.25–50 ng/mL was Y = 17.547x − 0.316 (R^2^ = 0.9999), where Y is the peak area of OTA and x is the OTA concentration (ng/mL). The retention time of OTA under the UPLC conditions used in this study was 5.979 min. The figure shows that both thermal–pressure treatment and fermentation caused a slight retention time shift and peak resolution loss. Baseline separation between OTA and other compounds was obtained in the extracts of GP samples that did not undergo thermal–pressure treatment or fermentation (Figure 1B,C). The reduced peak resolution in heat-treated and fermented GP samples (Figure 1D–G) might be caused by treatment-induced compositional changes in the GP extract, which may have a small and limited influence on the accuracy of OTA quantification.

### 3.2. Effect of Pressure Cooking on OTA Concentration and Polyphenol Composition in GP

**Figure 2:**
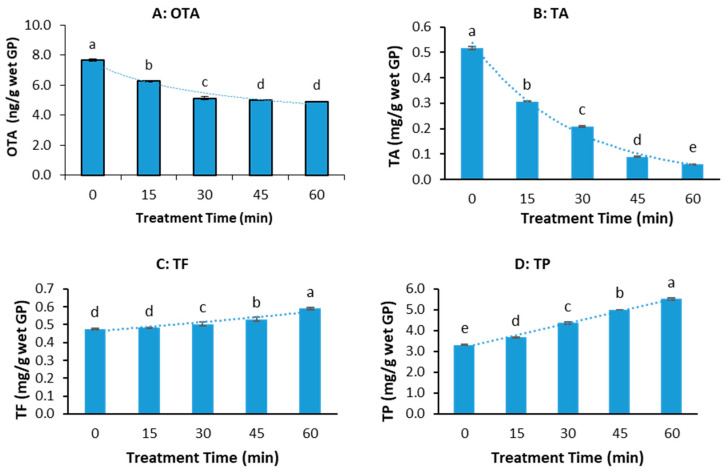
OTA and polyphenol contents of wet grape pomace (GP) at different pressure -cooking times. (**A**) OTA, (**B**) total anthocyanin (TA), (**C**) total flavonoid (TF), and (**D**) total polyphenol (TP). (The data bars with different letters are significantly different at *p* < 0.05).

Figure 2 shows the effect of pressure cooking alone on the OTA, TA, TF, and TP of GP. The OTA content in GP decreased significantly with cooking time in a power pattern (R^2^ = 0.9549), as shown in Figure 2A. Pressure cooking GP for 15 min decreased the OTA concentration by 18.11%. Extending the cooking time to 30, 40, and 45 min resulted in a 33.09%, 35%, and 36% OTA reduction, respectively, but the reduction of OTA from 45 min to 60 min was not significant.

Total anthocyanin (TA) decreased exponentially (R^2^ = 0.9853), dropping markedly by 39.7% to 88.1% from 15 to 60 min, suggesting near-complete degradation at prolonged cooking times (Figure 2B). In contrast, total flavonoid (TF) increased exponentially (R^2^ = 0.9093) with cooking time, although initial changes at 15 min were not significant (Figure 2C). Total polyphenol (TP) showed a linear increase (R^2^ = 0.9925), rising notably from 9.89% at 15 min to 40% at 60 min (Figure 2D). All observed changes were statistically significant (*p* < 0.05), highlighting the differential impact of pressure-cooking duration on specific polyphenol groups in grape pomace.

The results indicate that pressure cooking under home cooking conditions can reduce the OTA content in GP by up to 36% in a reasonable duration but cannot eliminate it. A more effective method needs to be discovered.

### 3.3. Effect of Pressure Cooking in the Presence of Acids on OTA Concentration and Polyphenol Composition in GP

**Figure 3:**
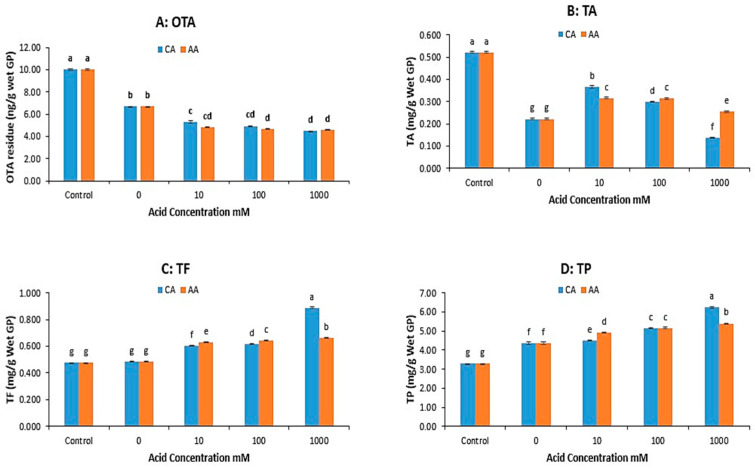
OTA and polyphenol concentrations in the grape pomace (GP) treated by pressure cooking (45 min) at different concentrations of citric acid (CA) and acetic acid (AA). (**A**) OTA, (**B**) total anthocyanin (TA), (**C**) total flavonoid (TF), (**D**) total polyphenol (TP). (The data bars with different letters are significantly different at *p* < 0.05).

As evidenced in Figure 2A, there was no significant decrease in OTA from 45 to 60 min. The pressure-cooking duration for the experiments on the impact of acid on the OTA and polyphenol contents of grape pomace was 45 min. Acetic and citric acid were used for acid treatment because they are widely used in food preparation and preservation. The results are shown in Figure 3.

Figure 3A shows the effect of types of acid and acid concentration on the OTA reduction of GP during pressure cooking. The addition of organic acids, specifically citric acid (CA) and acetic acid (AA), enhanced OTA reduction during the pressure cooking of grape pomace (GP), with higher reductions at increased acid concentrations—although no further reduction was observed beyond 100 mM. AA generally resulted in a slightly greater OTA reduction than CA.

For total anthocyanin (TA) in Figure 3B, acid addition significantly preserved levels compared to no acid treatment, though TA decreased as the acid concentration increased, especially at 1000 mM. AA preserved more TA than CA at higher acid concentrations, while CA performed better at the lowest concentration (10 mM). Figure 3C,D show that the total flavonoid (TF) and total polyphenol (TP) contents generally increased with higher acid concentrations. AA treatment usually retained higher TF content, except at the highest concentration (1000 mM), where CA preserved TF and TP better. Overall, incorporating organic acids (CA or AA) at concentrations of 10–100 mM during pressure cooking maximizes OTA reduction while minimizing polyphenol loss. Excessive acid (1000 mM) can exacerbate polyphenol degradation.

### 3.4. Effect of NaHCO_3_ and Pressure Cooking on OTA Concentration and Total Polyphenol Composition in GP

**Figure 4:**
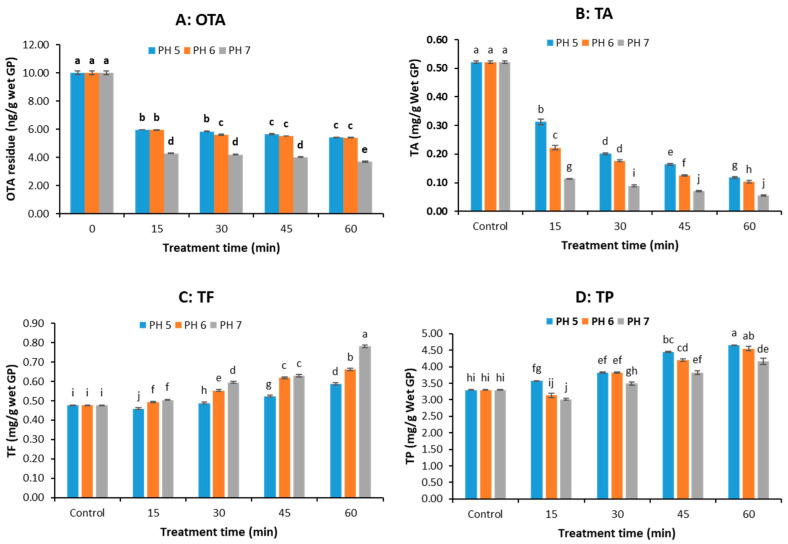
OTA and polyphenol concentrations of grape pomace (GP) treated by pressure cooking at pH 5, 6, and 7 (pH was adjusted with 100 mM NaHCO3 solution). (**A**) OTA, (**B**) total anthocyanin (TA), (**C**) total flavonoid (TF), (**D**) total polyphenol (TP). (The data bars with different letters are significantly different at *p* < 0.05).

The original pH of the grape pomace was about 4.5. In this study, the pH of GP was adjusted to 5, 6, and 7 using NaHCO_3_ and NaOH solutions before pressure cooking. Figure 4A demonstrates that the OTA concentration in GP samples decreased rapidly in the first 15 min then slowly at all pH levels. At same treatment time, there was no significant difference in OTA content in the GP samples pressure cooked at pH 5 and pH 6, except that at 30 min; however, there was a remarkable decrease in OTA content (up to 63.04% reduction at 60 min) when the pH increased from 6 to 7 (*p* < 0.05). The results suggest that a higher pH enhanced OTA degradation.

Figure 4B shows that both pH and pressure-cooking time significantly affect the TA content in the GP. TA decreased with both rising pH and longer cooking times, showing the greatest losses (up to 89.5%) at pH 7. At same treatment time, the TA content decreased with increasing pH value (*p* < 0.05). Thus, higher OTA reduction at elevated pH comes at the expense of anthocyanin preservation. The interaction between pH level and treatment time was significant (*p* ˂ 0.05), indicating the effect of treatment time on TA concentration varied with the pH level.

Figure 4C,D show that the TF and TP content of GP were significantly affected by pH and pressure-cooking time (*p* < 0.05). TF increased with higher pH and longer cooking times, showing maximum gains at pH 7. At same treatment time, the extractable/detectable TF increased significantly with pH (Figure 4C). TP generally decreased with higher pH but increased with cooking duration, though the retention was less effective compared to treatments with organic acids in Figure 3D.

Overall, pressure cooking at higher pH favored the degradation of OTA but not anthocyanin retention. Significant interaction between pH and treatment time (*p* ˂ 0.05) indicates the reduction in OTA concentration requires careful balancing of OTA reduction and polyphenol retention.

### 3.5. Effect of Fermentation Time on pH and Bacterial Populations in GP

**Figure 5:**
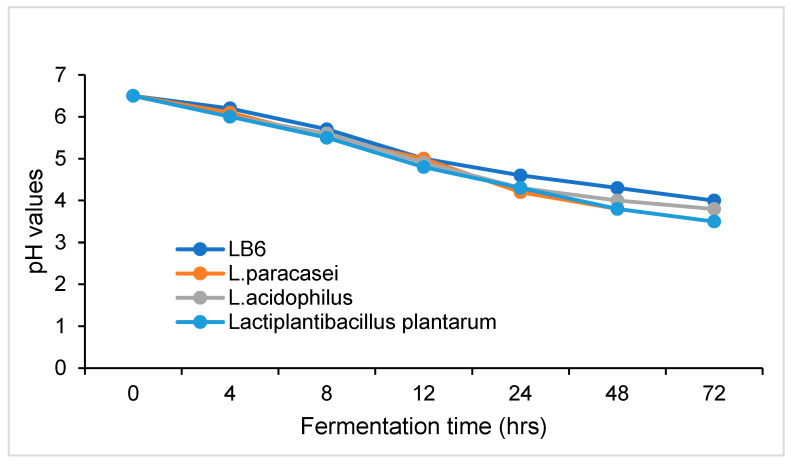
Changes in GP pH during fermentation with *lactic acid* bacteria.

The pH of GP was lowered to 6.02 from 6.5 due to pressure cooking, thus, cold, sterile NaOH (2M) was added to adjust the pH to 6.5, which is the preferred pH for LAB fermentation. Figure 5 shows the pH of GP decreased steadily with the fermentation time for all *Lactobacillus* strains used in this study. After 72 h of fermentation, the pH of GP was lower than that of untreated GP, indicating that lactic acid was produced during fermentation. The pH of all GP samples was about the same in the first 12 h of fermentation. At 24 h, the pH values of the GP samples fermented by *L. bulgaricus*, *Lc.paracasei*, *L.acidophilus*, and *Lp plantarum* were 4.6, 4.2, 4.3, and 4.3, respectively. Thereafter, the pH of the GP fermented with *L. bulgaricus* was slightly higher than that of GP fermented with other *LAB* strains for the same duration.

Two-way ANOVA showed that the viable bacterial counts in GP were significantly affected by fermentation time (*p* < 0.0001) and LAB strain (*p* < 0.0001) and that there was a significant interaction between these two variables (*p* < 0.0001). Table 1 shows that the viable bacterial counts in GP increased with fermentation time in the early stages, peaked between 8 and 12 h, and then declined steadily with time for all strains of LAB tested in this study. The fastest drop of viable bacteria count in GP samples was observed during fermentation with *Lp. Plantarum.* Post-hoc Tukey test indicates that the *L. acidophilus*-fermented GP had the highest viable bacterial count at all fermentation times. At 4 h, the *Lc. paracasei* count was the same as the *Lp. Plantarum* count but significantly higher than the *L. bulgaricus* count (*p* < 0.05). This might be caused by the lower initial viable bacterial count of *L. bulgaricus*, as shown at 0 h. From 12 to 72 h, the *L. bulgaricus* count increased faster than that for the other strains, reaching the same level as *Lp. plantarum* and significantly higher levels than *L. paracasei* (*p* < 0.05) at 24 h of fermentation. The viable bacteria counts at the end of fermentation (72 h) were significantly higher than or equal to their initial counts across all LAB strains.

### 3.6. Effects of Fermentation with Different Lactic Acid Bacteria Strains on the OTA in Grape Pomace

In this study, the main purpose of pressure cooking before fermentation was to inactivate the bacteria, molds, and yeasts that naturally exist in grape pomace, but this also reduced the OTA in GP by about 31% (Table 2). Two-way ANOVA shows that both LAB strains (*p* < 0.0001) and fermentation time (*p* < 0.0001) significantly affected the OTA content in the GP, and there was significant interaction between bacterial strain and fermentation time (*p* < 0.0001). Among the four strains, *L*. *bulgaricus* was the least effective; *L. bulgaricus* fermentation for 24–72 h resulted in only 6.7–13.82% OTA reduction. The other strains showed much higher and similar OTA reduction effectiveness and resulted in 62–66% OTA reduction in 24 h. For each bacterium strain, increasing the fermentation time led to a drastic reduction in OTA levels (*p* < 0.0001), particularly in the first 24 h of fermentation, except for *L. bulgaricus*, which showed a steady decrease in OTA during the elongated fermentation. There was a small but significant reduction in OTA from 24 to 72 h of fermentation for all strains (*p* < 0.05). Regression analysis of the data in Table 2 revealed that the OTA content linearly decreased with fermentation time in the first 24 h for all bacteria strains used in this study (R^2^ = 0.9826–0.9938). The Tukey test results show that among the three effective bacterium strains, *L. acidophilus*-fermented GP showed significantly lower OTA residue than GP fermented using the other strains (*p* < 0.05) for the same fermentation time, although the difference was small. The OTA contents in *L. paracasei-* and *Lp. plantarum*-fermented GP samples were not significantly different at most fermentation times but were obviously lower than that in *L. bulgaricus*-fermented GP (*p* < 0.05). The results indicate fermentation with *Lc. paracasei*, *L. acidophilus*, *or Lp. plantarum* following pressure cooking could result in near-complete degradation of OTA in 24 h under mild treatment conditions (42 °C). This is a promising and feasible method to detoxify OTA-contaminated GP for both food and feed applications.

### 3.7. Effects of Fermentation with Different Lactic Acid Bacteria on the Polyphenols in Grape Pomace

Table 3 shows that the lactic acid fermentation of GP with *Lactobacillus* significantly influenced the polyphenol composition of GP. The anthocyanin was mainly destroyed by pressure cooking before fermentation; the TA also decreased slightly but significantly with fermentation time (*p* < 0.05). Table 3 also shows that the TF contents of fermented GP were significantly affected by both fermentation time and bacterial strain (*p* < 0.05). The TF content in the GP increased with fermentation time for all *Lactobacillus* strains and reached its peak value at 24 h for *Lc. paracasei-*, *L. acidophilus*-, and *L. plantarum*-fermented GP and then gradually decreased as the fermentation time increased. For *L*. *bulgaricus-* and *Lp. plantarum*-fermented GP, the peak TF content appeared at 4 h and 12 h, respectively. In contrast, fermentation resulted in a time-dependent TP reduction for all bacterial strains. In the first 24 h, the TP decreased slowly, with *L. bulgaricus* as an exception. After 48 h, the TP content in the samples fermented with *L. acidophilus* and *L. plantarum* observed a larger decrease in TP compared to the other strains. Overall, the 24 h fermented GP samples had significantly higher extractable TF and TP contents than unfermented GP but almost no TA.

## 4. Discussion

This study found that pressure cooking grape pomace (GP) at 12 psig and 115 °C for 15–60 min reduced ochratoxin A (OTA) levels by 18.11–36.01%. This reduction is lower than the >50% reduction reported previously [15] following the autoclaving of GP at 121 °C (15 psig) for 30 min. This discrepancy strongly suggests that temperature is the predominant factor governing thermal OTA degradation efficiency. Our findings align with a consistent body of evidence across diverse food matrices. For instance, wet heating OTA in aqueous buffer showed degradation increasing from 1% to 86% as the temperature rose from 100 °C to 200 °C [20]. Similarly, the dry roasting of coffee beans yielded significantly better OTA reduction of 76.7–98.9% at 240 °C compared to only 8.2–53.6% at 180 °C [23]. The critical role of temperature over the moisture regime (dry vs. wet heating) is further emphasized by studies on wheat, where wet heating at 100 °C destroyed OTA while dry heating at the same temperature had no effect [26]. Thermal treatment facilitates the breakdown of OTA’s amide bond, leading to the formation of less toxic derivatives like ochratoxin α (OTα), 14-(R)-ochratoxin A (2′R-OTA), 14-decarboxy-ochratoxin A (DC-OTA), and ochratoxin α amide (OTα-amide) [21,27]. Consequently, while pressure cooking under the conditions tested effectively reduces microbial load and partially degrades OTA, thereby enhancing GP safety, it is insufficient for OTA elimination, particularly compared to higher-temperature processes like autoclaving. In real-world settings, most feed and food processors avoid very high temperatures (e.g., >160 °C) due to the high energy cost, technical challenges, and damage to product quality. Too much heat may cause nutrient loss and off-flavors, reduce bioactive compounds, and damage the structure of food and feed. Therefore, alternative detoxification methods with mild treatment conditions are preferred.

Regarding bioactive compounds, pressure cooking induced both positive and negative effects: a decrease in total anthocyanin (TA) but an increase in total flavonoid (TF) and total polyphenol (TP). The observed loss of TA is readily attributable to the heat instability of anthocyanins. It was reported that heating grape pomace at 60–125 °C for extended periods (more than 8 h) caused a considerable loss of anthocyanins (Khanal et al., 2010) [28]. The increase in TF and TP under the specific conditions employed in this study (pH ~4.5, in sealed container) could be attributed to (1) the relative stability of many phenolic compounds in acidic pH conditions [25,29,30]; (2) the minimized oxidative degradation of polyphenols due to low oxygen concentration in the sealed container filled with steam under pressure cooking [15]; and/or (3) the enhanced release of bound phenolic compounds caused by heat and pressure inducing the disruption of cell walls and cell membrane hydrophobic bonds, which increases the extractability polyphenols, as observed in studies using ohmic heating in wines [31] and high-temperature (160 °C) heating in brewers’ spent grain [32]. However, thermal treatment in the presence of air can induce various structural and chemical changes in polyphenols, such as isomerization, oxidative polymerization, and degradation, which not only affect quantification but also potentially alter their bioactivity [33].

This study demonstrated that the degradation of ochratoxin A (OTA) during the pressure cooking of grape pomace (GP) is significantly influenced by temperature, pH, and processing time. At the original acidic pH of GP (4.5), OTA reduction ranged from 18 to 36%, consistent with previous research [20]. The addition of citric acid (CA) or acetic acid (AA) to the GP enhanced the OTA detoxification efficacy of pressure cooking and led to a 46.9–55.2% OTA reduction. While effective, these reductions were slightly lower than the maximum reduction (61%) previously reported for GP treated solely with 10 mM organic acids (acetic, citric, lactic) at pH 2.0 and 37 °C for 24 h [15]. This divergence underscores the multifaceted nature of OTA degradation kinetics and highlights the critical parameters influencing efficacy. The differences in reduction levels may be due to the variations in acid concentration, treatment time, temperature, variety of grape pomace, and quantification methods used. This study employed ultra-performance liquid chromatography (UPLC), known for its consistency and accuracy, while the ELISA method used in the previous study has high variability due to the interference of GP polyphenols on OTA quantification, because polyphenols may bind to the antibody used in the ELISA, which prevents the antibody from binding OTA, though this has not been confirmed. Adjusting the GP pH to neutral (pH 7.0) using mild base (NaHCO_3_) increased OTA degradation to 63%, aligning with earlier findings that OTA degradation increases with higher pH [20,34]. However, strongly alkaline conditions risk forming toxic derivatives, such as open-lactone OTA (OP-OTA), which has higher toxicity than OTA [35]. Hence, a moderate alkaline condition (pH ≤ 7.0) is preferable.

In this study, the polyphenols in GP samples treated by pressure cooking only and the combination of pressure cooking with acids remained relatively stable compared to the pressure cooking with NaHCO_3_: this was due to the relative stability of polyphenols at acidic pH [31,32]. At low pH, polyphenols tend to remain in their protonated state, stabilizing specific structural features. For instance, anthocyanins predominantly exist as the red flavylium cation under strongly acidic conditions, a form that is relatively stable and responsible for the vibrant coloration in plant tissues [36]. This protonation minimizes the electron density on the aromatic rings, thereby reducing the propensity for oxidative reactions. However, extremely acidic environments can catalyze hydrolytic reactions, particularly in glycosylated polyphenols, leading to the cleavage of glycosidic bonds and altering their bioactivity [29]. Flavonoids are generally more stable due to the protonation of their phenolic hydroxyl groups. Further, the steam produced during pressure cooking may disrupt plant cell walls and release more phenolics [9]. However, heating at high pressure and temperature for a prolonged time also causes degradation of the released phenolics [37]. Thus, the heating time should not be too long. Anthocyanins were highly unstable at increased pH levels. As the pH increases, anthocyanins become more susceptible to degradation, potentially leading to plant material [38]. This study detected a loss of up to 89.48% of total anthocyanin during 60 min of pressure cooking at pH 7, whereas total flavonoids increased and total polyphenols generally decreased as pH increased. This pH-dependent polyphenol behavior is explained by the increased susceptibility of polyphenols to oxidative degradation under alkaline conditions due to hydroxyl group deprotonation and subsequent formation of reactive quinones and polymerization products [30,39,40]. Thermal–pressure treatments may affect the organoleptic properties of grape pomace, including color, aroma, and texture, which are critical for consumer acceptance in food or feed formulations. The effects can be desirable or detrimental. The desirable impacts include inactivating polyphenol oxidase and softening the texture of GP. The undesirable effects include heat induced browning/discoloration due to anthocyanin degradation and enhanced astringency due to the increased accessibility of TF and TP. The pressure cooking of GP may also generate a cooked flavor that may be preferred by some people but disliked by others. Though the use of citric and acetic acid stabilized the anthocyanins, it is also important to optimize the cooking time and temperature to limit the undesirable effect on these properties. In addition, polyphenols may function as pro-oxidants in formulated food and feed at high concentrations; therefore, the GP inclusion level also has to be optimized to minimize the possible negative impacts caused by high polyphenol concentrations.

This study demonstrated that the pressure cooking followed by fermentation of grape pomace (GP) using strains such as *L. acidophilus*, *L. paracasei*, and *Lactiplantibacillus plantarum* for 24–72 h reduced OTA levels by 97–98%, with fermentation alone accounting for over 60% of this reduction. However, fermentation with the strain *L. bulgaricus* only reduced OTA by 7–14%. These findings align with previous reported research results, where *L. rhamnosus* and *L. plantarum* degraded up to 97% and 95% of OTA, respectively, in MRS medium [41]. Adegoke et al. (2023) highlighted that different LAB strains have distinct capacities for mycotoxin reduction due to differences in cell-wall structure, metabolic pathways, and enzyme activities, thus emphasizing careful strain selection [42]. These differences can impact their effectiveness in transforming OTA into less toxic compounds and their OTA adsorption capability. The main purpose of pressure cooking before fermentation was to inactivate the bacteria, molds, and yeasts that naturally exist in grape pomace, as most food microorganisms can be inactivated by pressure cooking for 15–60 min [39].

Research has shown that fermentation using lactic acid bacteria (LAB) effectively reduces OTA by two different mechanisms: (1) physical adsorption of OTA onto bacterial cell walls, which can subsequently be removed by filtration post-fermentation, and (2) enzymatic conversion of OTA into less toxic OTα and phenylalanine by the enzymes secreted during fermentation [43,44,45]. In the present study, the observed OTA reduction in fermented GP was primarily attributed to enzymatic degradation rather than physical adsorption, since OTA extraction included bacterial cells and GP residue, although adsorption might have partially contributed to the decrease in OTA. As shown in Table 1, LAB counts reached >9 log CFU/g across all strains. However, *L. bulgaricus* achieved only ~45% OTA reduction, despite similar viable counts, while *L. acidophilus*, *Lc. paracasei*, and *Lp. plantarum* achieved 98% OTA reduction at a fermentation time of 72 h (Table 2). This indicates that OTA reduction is not directly correlated to bacterial growth and the viable LAB counts did not predict detoxification efficacy. The data suggest that the primary mechanism of OTA reduction in GP by *L. bulgaricus* is different than that by other LAB strains. While the primary mechanism of OTA reduction by *L. acidophilus*, *Lc. paracasei*, and *Lp. plantarum* is enzymatic degradation, adsorption might be the primary mechanism for *L. bulgaricus*. It may also be possible that the OTA-degrading enzymes secreted by *L. bulgaricus* cells were less active than those produced by the other LAB strains used in this study, thus taking a longer time to achieve high OTA reduction.

It is notable that the viable bacterial counts in GP increased with fermentation time in the first 12–24 h. The counts then declined but were still higher than the initial counts for all LAB strains (Table 1). The decreased growth of LAB bacteria might be due to the depletion of nutrients and the decrease in pH as fermentation time increased. It is well known that plant polyphenols have antimicrobial activity, including against lactic acid bacteria. However, the inhibitory effects of GP polyphenols on LAB growth were not observed in this study. Previous studies also found that GP polyphenols can act as prebiotics, promoting the growth and activity of certain *Lactobacillus* strains, such as *Lp plantarum*, *L. casei*, and *L. bulgaricus*, but inhibiting the growth of *L. acidophilus* and *Limosilactobacillus vaginalis*. Grape seed extract decreased the growth of most *Lactobacillus* bacteria [46,47,48]. The results of this study support the previous findings, although more research is needed to reveal how GP polyphenols influence the viability of LAB.

The pH of GP fermented with different strains of *Lactobacillus* differed at the same fermentation time, particularly after 24 h (Figure 4). This pH variation may be caused by the significant difference in sugar metabolic rates among different strains of LAB. The higher pH of *L. bulgaricus*-fermented GP may be the result of the slow metabolism of sucrose by *L. Bulgaricus*, because *L. Bulgaricus* prefers lactose and galactose as its substrate [49], while other *Lactobacillus* strains exhibit a broader range of carbohydrate utilization abilities [50,51].

Lactic acid fermentation significantly affects the polyphenol profile of grape pomace (GP), generally enhancing the extractability and bioavailability of phenolic compounds, though the outcomes vary with fermentation conditions, bacterial strains, and grape varieties [46,47]. This study demonstrated the dual effects of fermentation on the polyphenols of grape pomace (Table 3). The total flavonoid (TF) content increased within the first 24 h of fermentation for all tested *Lactobacillus* strains, followed by a gradual decline, except in the *LB6* strain, where TF levels remained relatively stable. In contrast, total anthocyanin (TA) and total polyphenol (TP) decreased progressively throughout fermentation, reflecting the potential degradation of these compounds. These findings are consistent with previous reports [52,53], where a decrease in polyphenol content was observed during one-month ensiling of *Vitis vinifera* cv. Nero di Troia GP, especially when fermented with *Lp. plantarum*. However, other studies have reported contradictory results. Balea et al. (2018) found that a 20-day fermentation of *Fetească neagră* GP increased polyphenol levels [46], while Campanella et al. (2017) reported either no change or a decrease after 24 h of fermentation, depending on the LAB strain used [45].

LAB contributes to polyphenol transformation by secreting enzymes such as β-glucosidases, phenolic acid decarboxylases, and esterases, which cleave glycosylated or esterified polyphenols into more bioavailable aglycones [53]. The enzymatic capacity is strain-dependent, influencing the degree and type of polyphenol transformation. Extended fermentation may enhance the release of phenolic acids and flavonoids (e.g., gallic, caffeic, ferulic acids, and quercetin) due to enzymatic breakdown of the cell wall matrix, but it also increases the risk of degradation of labile compounds, such as anthocyanins [54]. As shown in Table 3, the loss of anthocyanins was primarily caused by pressure cooking at pH 6.5 for 45 min; thus, decreasing the pH and shortening the pressure-cooking time could preserve more anthocyanins. Meanwhile, selecting a more acid-tolerant bacterium strain, such as *L. acidophilus*, is also important for achieving high OTA reduction and anthocyanin retention because it allows the fermentation to be conducted at a relatively lower pH. The observed changes in polyphenol levels during thermal–pressure treatment and fermentation are not merely quantitative but may also impact the functional health benefits of GP. An increase in total flavonoids and total phenolics suggests improved antioxidant capacity and potential anti-inflammatory or antimicrobial effects; however, these beneficial effects may be counteracted by the loss of anthocyanins.

## 5. Conclusions

This study found that thermal–pressure treatment of GP under typical home pressure-cooking conditions, without pH adjustment, achieved only about 35% OTA reduction, which was statistically significant but insufficient for ensuring food safety. Pressure cooking had mixed effects on polyphenols: while it reduced TA, it increased detectable TF and TP. The addition of acids during pressure cooking improved OTA reduction to 55% and enhanced polyphenol retention, especially TA. Conversely, using NaHCO_3_ raised OTA reduction to 63% at pH 7 but caused greater anthocyanin degradation, with minimal effects on TF and TP. Combining pressure cooking with lactic acid bacteria (LAB) fermentation achieved up to 98% OTA reduction, though it also had varied impacts on polyphenol content. These findings highlight that pressure cooking followed by LAB fermentation is a promising method for detoxifying OTA-contaminated GP and potentially other mycotoxin-contaminated grains. In addition to OTA reduction and polyphenol changes during pressure cooking and subsequent fermentation, this combined process can destroy pathogens, including mycotoxin-producing fungi such as *Aspergillus ochraceus* and *Penicillium verrucosum*. Pressure cooking at 115 °C will likely contribute to fungal inactivation, thereby preventing further OTA production. Similarly, LAB fermentation can suppress fungal growth via acidification, competition, and production of antifungal compounds such as organic acids. Further, the fermented GP could serve as a probiotic source due to its high concentration of LAB. Thus, the combined pressure cooking and LAB fermentation can enhance the safety and health benefits of grape pomace.

The limitations of this study are as follows: (1) the OTA degradation products, such as ochratoxin α (OTα), were not quantified due to the lack of analytical standards, which makes it uncertain whether the OTA-related toxicity of treated GP is really reduced, (2) the grape pomace was artificially spiked with OTA solution rather than naturally contaminated, which may not fully represent the natural OTA distribution in GP, and (3) the organoleptic properties of GP after detoxification were not assessed, which is important for feed and food applications. Future research will explore the OTA degradation products during pressure cooking and fermentation using LC/MS or LC/MS-MS, the effects of pressure cooking and fermentation on the individual polyphenols or polyphenol profile of GP using UPLC/MS-MS, and the toxicity of pressure-cooked and fermented GP spiked with OTA in comparison with the OTA-spiked but untreated GP using a poultry model.

## Figures and Tables

**Table 1:** Bacteria population in GP during fermentation (LogCFU/mL).

Fermentation Time (h)	*Lc. paracasei*	*L. acidophilus*	*Lp. plantarum*	*L. bulgaricus*
**0**	8.27 ± 0.01 ^fB^	8.46 ± 0.01 ^gA^	8.45 ± 0.01 ^fA^	8.00 ± 0.01 ^gC^
**4**	8.87 ± 0.08 ^dB^	9.12 ± 0.02 ^eA^	8.95 ± 0.03 ^deB^	8.42 ± 0.01 ^fC^
**8**	9.27 ± 0.02 ^aC^	9.44 ± 0.01 ^bA^	9.31 ± 0.01 ^aB^	9.27 ± 0.01 ^bC^
**12**	9.18 ± 0.03 ^bC^	9.47 ± 0.01 ^aA^	9.19 ± 0.02 ^bC^	9.32 ± 0.01 ^aB^
**24**	9.02 ± 0.04 ^cC^	9.38 ± 0.01 ^cA^	9.07 ± 0.03 ^cC^	9.24 ± 0.01 ^cB^
**48**	8.87 ± 0.03 ^dD^	9.22 ± 0.02 ^dA^	8.99 ± 0.04 ^dC^	9.10 ± 0.02 ^dB^
**72**	8.71 ± 0.03 ^eC^	9.03 ± 0.03 ^fA^	8.41 ± 0.01 ^gD^	8.88 ± 0.03 ^eB^

Note: In the same column, data with different lowercase superscripts are significantly different at *p* < 0.05. In the same row, data with different uppercase superscripts are significantly different at *p* < 0.05.

**Table 2:** Effect of fermentation with four lactic acid bacteria strains on OTA concentration in GP.

	*L. bulgaricus*		*Lc. paracasei*		*L. acidophilus*		*Lp. plantarum*	
Fermentation Time (h)	OTA (ng/g)	Reduction (%)	OTA (ng/g)	Reduction (%)	OTA (ng/g)	Reduction (%)	OTA (ng/g)	Reduction (%)
UNT	50.00 ± 0.41 ^aA^	0	50.00 ± 0.41 ^aA^	0	50.00 ± 0.41 ^aA^	0	50.00 ± 0.41 ^aA^	0
0	34.51 ± 0.48 ^bA^	30.99	34.74 ± 0.39 ^bA^	30.99	34.51 ± 0.48 ^bA^	30.99	34.51 ± 0.48 ^bA^	30.99
4	33.81 ± 0.16 ^bA^	32.38	28.00 ± 0.26 ^cC^	44.37	26.57 ± 0.52 ^cD^	46.86	28.13 ± 0.52 ^cB^	43.75
8	33.20 ± 0.20 ^dA^	33.6	22.37 ± 0.32 ^dB^	55.56	20.96 ± 0.59 ^dC^	58.09	20.92 ± 0.62 ^dC^	58.16
12	32.73 ± 0.30 ^dA^	34.55	15.76 ± 0.24 ^eB^	68.7	15.47 ± 0.23 ^eB^	69.05	14.99 ± 0.07 ^eC^	70.02
24	31.15 ± 0.24 ^eA^	37.7	1.52 ± 0.02 ^fB^	96.98	1.14 ± 0.03 ^fD^	96.7	2.45 ± 0.13 ^fC^	92.89
48	27.96 ± 0.58 ^fA^	41.81	1.44 ± 0.06 ^fgC^	97.14	1.01 ± 0.03 ^hD^	97.98	2.40 ± 0.47 ^gB^	95.2
72	27.59 ± 0.14 ^fgA^	44.81	1.17 ± 0.03 ^hC^	97.68	1.00 ± 0.03 ^hD^	97.1	1.37 ± 0.02 ^ghB^	96.02

Note: In the same column, data with different superscripts are significantly different at a 5% significance level. In the same row, data with different uppercase superscripts are significantly different at *p* < 0.05. UNT—OTA-spiked grape pomace sample without pressure cooking and fermentation; “0” time points—grape pomace sample spiked with OTA and pressure cooked.

**Table 3:** Effects of fermentation time and bacterial strain on polyphenol composition in GP.

Fermentation Time (h)	*Lc. paracasei*	*L. acidophilus*	*Lp. plantarum*	*L. bulgaricus*
TA (mg/g)				
UNT	0.49 ± 0.01 ^a^	0.49 ± 0.01 ^a^	0.49 ± 0.01 ^a^	0.49 ± 0.01 ^a^
0	0.07 ± 0.00 ^b^	0.07 ± 0.00 ^b^	0.07 ± 0.00 ^b^	0.07 ± 0.00 ^b^
4	0.06 ± 0.00 ^c^	0.06 ± 0.00 ^c^	0.06 ± 0.00 ^c^	0.06 ± 0.00 ^c^
8	0.06 ± 0.00 ^c^	0.06 ± 0.00 ^c^	0.06 ± 0.00 ^c^	0.06 ± 0.00 ^c^
12	0.06 ± 0.00 ^c^	0.06 ± 0.00 ^c^	0.06 ± 0.00 ^c^	0.06 ± 0.00 ^c^
24	0.06 ± 0.00 ^c^	0.05 ± 0.00 ^d^	0.05 ± 0.00 ^d^	0.06 ± 0.00 ^c^
48	0.06 ± 0.00 ^c^	0.05 ± 0.00 ^d^	0.05 ± 0.00 ^d^	0.05 ± 0.00 ^d^
72	0.05 ± 0.00 ^d^	0.05 ± 0.00 ^d^	0.05 ± 0.00 ^d^	0.05 ± 0.00 ^d^
TF (mg/g wet GP)				
UNT	0.47 ± 0.00 ^h^	0.47 ± 0.00 ^h^	0.47 ± 0.00 ^h^	0.47 ± 0.00 ^f^
0	0.62 ± 0.01 ^g^	0.62 ± 0.01 ^f^	0.62 ± 0.01 ^f^	0.62 ± 0.01 ^d^
4	0.68 ± 0.00 ^eB^	0.69 ± 0.01 ^e^	0.65 ± 0.01 ^eC^	0.82 ± 0.01 ^aA^
8	0.76 ± 0.01 ^cB^	0.78 ± 0.01 ^cA^	0.74 ± 0.01 ^cC^	0.76 ± 0.01 ^bB^
12	0.79 ± 0.01 ^bB^	0.82 ± 0.02 ^bA^	0.79 ± 0.01 ^bB^	0.76 ± 0.01 ^bC^
24	0.81 ± 0.00 ^aB^	0.91 ± 0.01 ^aA^	0.82 ± 0.01 ^aB^	0.65 ± 0.00 ^cC^
48	0.70 ± 0.01 ^dB^	0.75 ± 0.00 ^dA^	0.71 ± 0.02 ^dB^	0.62 ± 0.01 ^dC^
72	0.65 ± 0.00 ^fA^	0.58 ± 0.01 ^gB^	0.49 ± 0.00 ^gC^	0.57 ± 0.01 ^eB^
TP (mg/g wet GP)				
UNT	3.22 ± 0.01 ^e^	3.22 ± 0.01 ^f^	3.22 ± 0.01 ^g^	3.22 ± 0.01 ^g^
0	3.79 ± 0.05 ^a^	3.79 ± 0.05 ^a^	3.79 ± 0.05 ^a^	3.79 ± 0.05 ^a^
4	3.73 ± 0.01 ^abB^	3.75 ± 0.00 ^bA^	3.69 ± 0.04 ^bBC^	3.65 ± 0.04 ^bCD^
8	3.70 ± 0.00 ^bB^	3.73 ± 0.01 ^bcA^	3.67 ± 0.03 ^bcC^	3.60 ± 0.01 ^bcD^
12	3.67 ± 0.02 ^cB^	3.71 ± 0.01 ^cA^	3.61 ± 0.01 ^dC^	3.51 ± 0.04 ^dD^
24	3.60 ± 0.01 ^dB^	3.67 ± 0.03 ^dA^	3.53 ± 0.03 ^eC^	3.41 ± 0.02 ^eD^
48	3.24 ± 0.05 ^eC^	3.27 ± 0.03 ^eBC^	3.40 ± 0.00 ^fA^	3.31 ± 0.01 ^fB^
72	3.01 ± 0.03 ^fB^	2.68 ± 0.00 ^gC^	2.69 ± 0.02 ^hC^	3.20 ± 0.03 ^gA^

Note: In the same column, data with different lowercase superscripts are significantly different at *p* < 0.05. In the same row, data with different uppercase superscripts are significantly different at *p* < 0.05. UNT—OTA-spiked grape pomace sample without pressure cooking and fermentation; “0” time points—grape pomace sample spiked with OTA and pressure cooked.

## Data Availability

The original contributions presented in this study are included in the article. Further inquiries can be directed to the corresponding author.

## Abbreviations

The following abbreviations are used in this manuscript:

AA	Acetic Acid
CA	Citric Acid
DI	Deionized Water
GP	Grape Pomace
HCL	Hydrochloric Acid
LAB	Lactic Acid Bacteria
NaHCO_3_	Sodium Bicarbonate
NaOH	Sodium Hydroxide
OD	Optical Density
OTA	Ochratoxin A
OTα	Ochratoxin alpha
TA	Total Anthocyanin
TF	Total Flavonoid
TP	Total Polyphenol
UPLC	Ultra-Performance Liquid Chromatography
